# Antimicrobial resistance in ICU endotracheal aspirates: Evidence to support stewardship in Bangladesh

**DOI:** 10.6026/973206300221784

**Published:** 2026-03-31

**Authors:** Sanjida Khondakar Setu, Abu Naser Ibne Sattar, Towfique Hasan Firoz, Sanjar Taufiq

**Affiliations:** 1 Department of Microbiology & Immunology, Bangladesh Medical University, Shahbag, Dhaka, Bangladesh; 2 Department of Radiotherapy, National Institute of Cancer Research and Hospital, Mohakhali, Dhaka, Bangladesh; 3 Department of Biotechnology, BRAC University, Dhaka, Bangladesh

**Keywords:** Ventilator-associated respiratory infections (VARI), endotracheal aspirate, evidence-based antimicrobial resistance, antimicrobial stewardship, bacterial pathogens in VAP

## Abstract

Ventilator-associated respiratory infections are a major cause of morbidity and mortality in mechanically ventilated ICU patients. Therefore, it is of interest to analyze endotracheal aspirates collected from adult ICU patients at Bangladesh Medical University between July 2024 to June 2025. Among 111 samples examined, 26.1% showed significant bacterial growth. Gram-negative *bacilli* predominated (96.6%) among the isolates. The most frequent pathogen was *Pseudomonas aeruginosa* (58.6%), followed by *Acinetobacter baumannii*, *Klebsiella pneumoniae* and *Escherichia coli*. High resistance was observed to third-generation cephalosporins, fluoroquinolones and aminoglycosides. Reduced susceptibility to carbapenems was also detected among *P. aeruginosa* and *A. baumannii* isolates. All Gram-negative isolates remained susceptible to colistin. The single *Staphylococcus aureus* isolate was susceptible to vancomycin. Thus, we show the predominance of multidrug-resistant Gram-negative pathogens in ventilated ICU patients. Institution-specific susceptibility data are essential to guide rational empiric therapy and support antimicrobial stewardship in resource-limited ICUs.

## Background:

Ventilator-associated respiratory infections (VARI), including ventilator-associated tracheobronchitis (VAT) and ventilator-associated pneumonia (VAP), remain major complications in critically ill patients receiving mechanical ventilation. These infections are associated with prolonged mechanical ventilation, longer ICU and hospital stays and increased morbidity. Multicenter prospective cohort studies have reported significantly longer ventilation durations and hospital stays in patients with VARI compared with non-infected patients [1]. Recent evidence also shows that VAP affects a considerable proportion of ventilated ICU patients and increases healthcare utilization and antibiotic exposure [2]. Placement of an endotracheal tube disrupts normal airway defenses by impairing mucociliary clearance and facilitating micro aspiration of contaminated secretions. This process promotes bacterial colonization and biofilm formation on the tube surface, creating a reservoir for pathogens [3]. Gram-negative *bacilli* such as *Pseudomonas aeruginosa*, *Acinetobacter baumannii*, *Klebsiella pneumoniae* and *Escherichia coli*, along with *Staphylococcus aureus*, are the predominant causes of VAP worldwide. Stoian *et al.* reported Acinetobacter, Pseudomonas, Klebsiella and *Staphylococcus aureus* as the most common VAP pathogens in a recent ICU cohort [4]. Another study similarly identified *Pseudomonas aeruginosa*, *Acinetobacter baumannii* and *Klebsiella pneumoniae* as dominant Gram-negative pathogens in VAP [5]. Ventilator-associated pneumonia contributes substantially to adverse outcomes in critically ill patients. Mortality rates may reach up to 50%, increasing to nearly 76% when infections are caused by multidrug-resistant pathogens [6]. The situation is further complicated by the rising prevalence of carbapenem-resistant Gram-negative bacteria in ICUs. An ICU study by Karget *et al.* (2025) showed that infections caused by carbapenem-resistant Gram-negative bacteria were strongly associated with treatment failure and higher mortality [7]. Early identification of causative organisms and their antimicrobial susceptibility patterns is therefore essential for optimal management. Timely initiation of appropriate antimicrobial therapy has consistently been associated with improved survival in severe infections, including VAP [6]. However, locally generated microbiological data on pathogen distribution and resistance patterns remain limited in many settings. This lack of data restricts evidence-based empirical antibiotic selection and weakens antimicrobial stewardship efforts in ICUs [8]. The epidemiology of ventilator-associated pneumonia also varies widely across regions and healthcare settings. Adrião *et al.* (2025) reported that the incidence of VAP ranges from 5% to 40% depending on geographic location and diagnostic criteria [9]. Therefore, it is of interest to update microbiological evidence on pathogens recovered from endotracheal aspirates of mechanically ventilated ICU patients in Bangladesh.

## Methods:

## Study design and setting:

A retrospective observational study was conducted using microbiological data from endotracheal tube (ETT) aspirate specimens of patients receiving mechanical ventilation in the Intensive Care Units (ICUs) of Bangladesh Medical University between July 2024 and June 2025.

## Study population:

Adult patients (≥18 years) admitted to the ICU, intubated and mechanically ventilated for at least 48 hours and with clinical suspicion of ventilator-associated respiratory infection were included. Patients ventilated for less than 48 hours and specimens yielding only commensal flora or contaminants were excluded. This approach enabled assessment of pathogen distribution and antimicrobial susceptibility patterns relevant to ventilator-associated infection surveillance and antibiotic stewardship.

## Specimen collection and processing:

Following extubation, the distal 2-3 cm of each ETT was aseptically excised using sterile scissors and transferred to a sterile container. In the microbiology laboratory, the internal lumen was irrigated with 0.5 mL of sterile saline and the eluate was collected for analysis.

## Mechanical liquefaction and concentration:

Specimens were vortexed for 1 minute to ensure homogenization, then centrifuged at 2000 x g for 10 minutes. The supernatant was discarded and the sediment was re-vortexed to obtain a uniform suspension for culture and microscopic examination.

## Semi-quantitative culture:

Aliquots of 10 µL and 1 µL of the processed specimen were inoculated onto blood agar and MacConkey agar using calibrated loops and streaked across three consecutive sectors. Plates were incubated aerobically at 37°C for 24 hours. Growth was graded as rare, light, moderate, or heavy. Moderate or heavy growth in at least two consecutive sectors was considered clinically significant and included in the analysis.

## Identification and antimicrobial susceptibility testing:

Bacterial identification and antimicrobial susceptibility testing were performed using VITEK® 2 Compact identification and AST cards (N280 and N281) according to the manufacturer's instructions.

## Quality control:

Quality control was ensured using standard reference strains of *Escherichia coli*, *Klebsiella pneumoniae* and *Staphylococcus aureus*, tested at 15-day intervals throughout the study period.

## Ethical approval:

This study utilized anonymized microbiology laboratory data collected as part of routine diagnostic services. No patient identifiers or clinical information were accessed. In accordance with the institutional policies, ethical approval and informed consent were exempted as the study involved secondary analysis of de-identified data. This exemption is per the IVI IRB SOP D-RB-4-003.

## Results and Discussion:

In this retrospective analysis of endotracheal aspirates from mechanically ventilated ICU patients, multidrug-resistant (MDR) Gram- negative *bacilli* predominated. *Pseudomonas aeruginosa* constituted the majority of isolates (58.6%), followed by *Acinetobacter baumannii*, *Klebsiella pneumoniae* and *Escherichia coli*, while only a single Gram-positive organism, *Staphylococcus aureus* and was isolated. (Figure 1 - see PDF). The spectrum of culture positivity and Gram reaction is summarized in Table 1 (see PDF). The overall culture positivity rate was 26.1%. High resistance was observed against third-generation cephalosporins, fluoroquinolones and aminoglycosides agents commonly used for empiric therapy in ICUs. In contrast, colistin demonstrated complete activity against all Gram-negative isolates and vancomycin retained full efficacy against the lone *Staphylococcus aureus* isolate. The predominance of Gram-negative *bacilli* (96.5%) observed in this study is consistent with contemporary regional patterns of ventilator-associated respiratory infections, in which Gram-negative organisms constitute the majority of isolates, with *Acinetobacter baumannii*, *Pseudomonas aeruginosa* and *Klebsiella pneumoniae* species most frequently identified. Recent ICU-based VAP studies similarly report a predominance of Gram-negative pathogens, particularly *Acinetobacter baumannii*, *Klebsiella pneumoniae* and *Pseudomonas aeruginosa*, among leading causative organisms [10]. Comparable findings have also been documented in Oman, where ICU surveillance data demonstrate Gram-negative organisms as the most prevalent isolates and reveal a substantial burden of multidrug-resistant organisms, underscoring the importance of locally generated microbiological data to guide empirical therapy and antimicrobial stewardship strategies [11]. Studies from India further demonstrate comparable dominance of Gram-negative organisms particularly *Acinetobacter baumannii*, *Klebsiella pneumoniae* and *Pseudomonas aeruginosa* in endotracheal and tracheal aspirates [12]. Collectively, these findings highlight the substantial burden of MDR Gram-negative pathogens in ventilator-associated infections across South Asia and neighboring regions.

Our findings demonstrated a higher proportion of *Pseudomonas aeruginosa*, with relatively fewer *Acinetobacter baumannii*. Contemporary literature from Asia continues to describe *Acinetobacter baumannii* among the most frequently identified Gram-negative VAP pathogens, followed by *Pseudomonas aeruginosa* and *Klebsiella pneumoniae* [13]. Such differences in pathogen distribution may reflect variation in ICU ecology, ventilator circuit management, environmental contamination (including water sources), patient case-mix, duration of mechanical ventilation and cumulative antimicrobial exposure, all of which influence VAP microbiology [14]. Antimicrobial susceptibility testing revealed alarmingly high resistance to third-generation cephalosporins, ciprofloxacin and gentamicin across *Pseudomonas aeruginosa*, *Acinetobacter baumannii*, *Klebsiella pneumoniae* and *Escherichia coli* (Table 2 - see PDF). These findings mirror other regional reports. In a recent Bangladeshi ICU study using endotracheal tube (ETT) derived tracheal aspirates, Acinetobacter spp. demonstrated very high resistance to both ceftriaxone (98.9%) and gentamicin (92.5%), with over 90% of isolates exhibiting multidrug resistance and marked resistance to carbapenems indicating limited utility of these agents for empiric coverage in ventilated patients [15]. Our study showed high fluoroquinolone resistance (64.7-100%) and similar studies of multidrug-resistant organisms report frequent resistance to third-generation cephalosporins, fluoroquinolones and aminoglycosides often above 65% which is consistent with our findings [16]. Comparable resistance patterns to cephalosporins, aminoglycosides and fluoroquinolones are also consistently documented in global ventilator- associated pneumonia [17]. Carbapenem resistance in our study was high in *Klebsiella pneumoniae* (66.6%) and *Escherichia coli* (50%), but lower in *Acinetobacter baumannii* (33.3%). In contrast, a multicenter study from Oman reported the highest resistance in *Acinetobacter baumannii* (80.4%), followed by *Klebsiella pneumoniae* (46.4%) and *Pseudomonas aeruginosa* (29.9%) [18], while a South Indian study found lower resistance in *Acinetobacter baumannii* (48.0%) and *Klebsiella pneumoniae* (38.6%) and markedly lower rates in *Escherichia coli* (12.1%) [19]. These differences highlight substantial geographic and setting-specific variation in carbapenem resistance [18]. Notably, colistin retained complete activity against all Gram-negative isolates and vancomycin remained fully effective against the lone *Staphylococcus aureus* isolate, consistent with contemporary ICU susceptibility data [5, 20 and 21]. The findings of this study are in line with the concerns outlined in the Report on Antimicrobial Resistant Surveillance Bangladesh, 2024, which underscores the increasing burden of AMR in Bangladesh. Although the national report presents a broader surveillance perspective, our study contributes specific evidence from ICU endotracheal aspirates, thereby complementing national data with institution-based microbiological insights [22]. These findings may help inform local antimicrobial stewardship and empirical treatment practices in in mechanically ventilated ICU patients in Bangladesh.

## Limitations:

The retrospective design limited clinical correlation with outcomes and prior antibiotic exposure. Use of routine culture methods may have underestimated fastidious, anaerobic and fungal pathogens. The small sample size limits generalizability, highlighting the need for larger prospective studies with comprehensive microbiological and clinical assessment.
